# Supplementary material for: A Comprehensive Assessment of Ultraviolet-Radiation-Induced Mutations in Flammulina filiformis Using Whole-Genome Resequencing
Source: J Fungi (Basel). 2024 Mar 20;10(3):228. doi: 10.3390/jof10030228 (PMC10971301; doi:10.3390/jof10030228)
Supplement: Supplementary file 1 [file jof-10-00228-s001.zip › Supplementary Material S8/KEGG annotation/out/64381550635650.os/KO/out_map/map04340.html]

KEGG PATHWAY: Hedgehog signaling pathway - Reference pathway


|  |  |
| --- | --- |
| **Hedgehog signaling pathway - Reference pathway** |  |

[
Pathway menu
| Organism menu
| Pathway entry
| Show description
| User data mapping
]

The Hedgehog (Hh) family of secreted signaling proteins plays a crucial role in development of diverse animal phyla, from Drosophila to humans, regulating morphogenesis of a variety of tissues and organs. Hh signaling is also involved in control of stem cell proliferation in adult tissues and aberrant activation of the Hh pathway has been linked to multiple types of human cancer. Members of the Hh family bind to patched (ptc), thus releasing smoothened (smo) to transduce a signal. Transcriptional activation occurs through the GLI family of proteins resulting in activation of target genes.

|  |  |
| --- | --- |
| Reference pathway | 100% |
